# Supplementary material for: Chimeric chromosome landscapes of human somatic cell cultures show dependence on stress and regulation of genomic repeats by CGGBP1
Source: Oncotarget. 2022 Jan 17;13:136–55. doi: 10.18632/oncotarget.28174 (PMC8765472; doi:10.18632/oncotarget.28174)
Supplement: Supplementary file 1 [file oncotarget-13-28174-s001.pdf]

Chimeric chromosome landscapes of human somatic cell cultures show dependence on stress and regulation of genomic repeats by CGGBP1

SUPPLEMENTARY MATERIALS

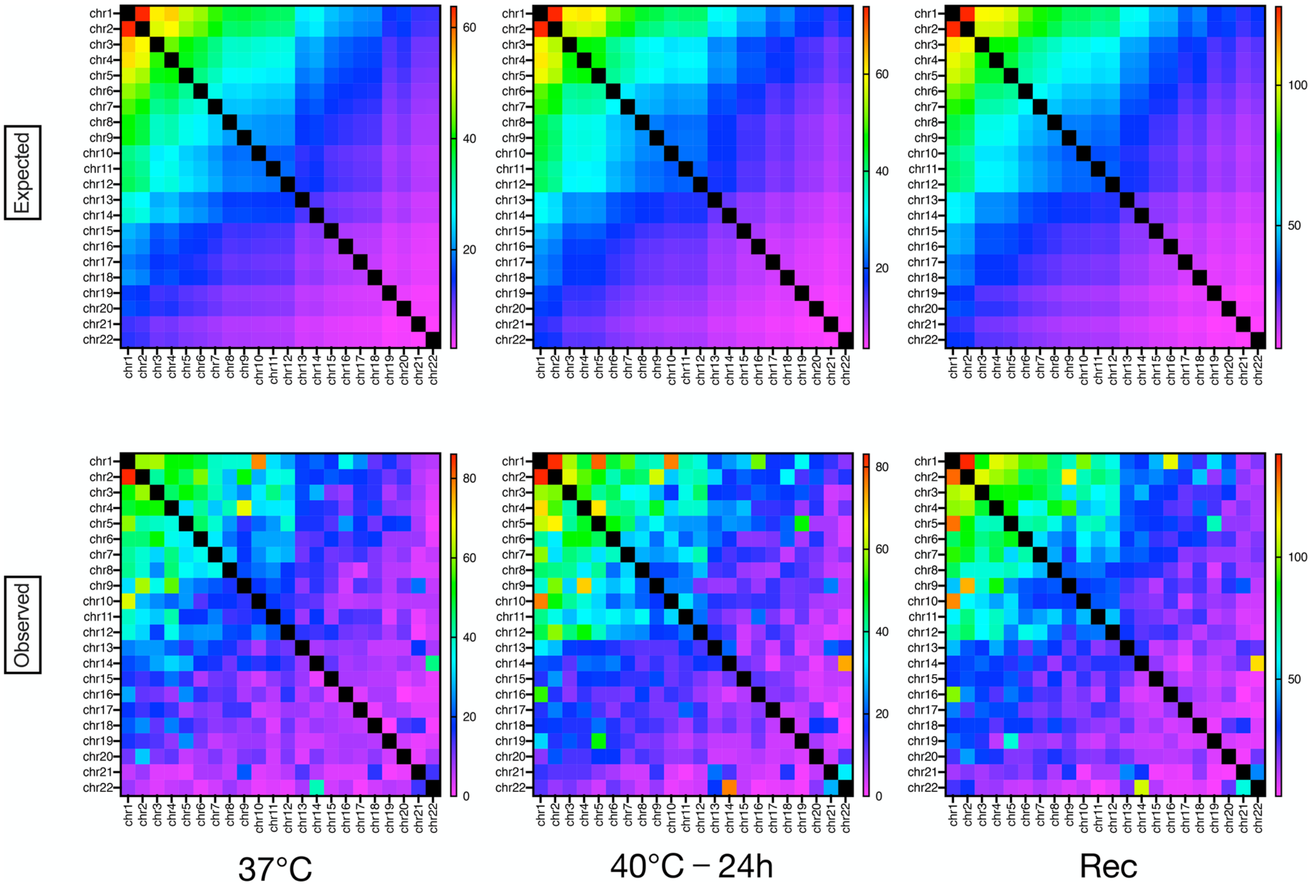

Supplementary Figure 1: The dependence of chimeric chromosomal DNA in GM02639 cells on chromosomal lengths indicate the randomness of the events: The expected chimeric events depict the distribution profiles if all the events were distributed evenly on the different autosomes proportional to their lengths. The observed profiles show a strong dependence of chimeric event occurrence on chromosomal lengths. Some exceptions were observed, which were however inconsistent, again indicating no consistent preference for specific interchromosomal chimeric events. For the calculation of these profiles, the A-U-B events were used non-directionally such that the profiles do not represent A-U-B events differently from the B-U-A events.

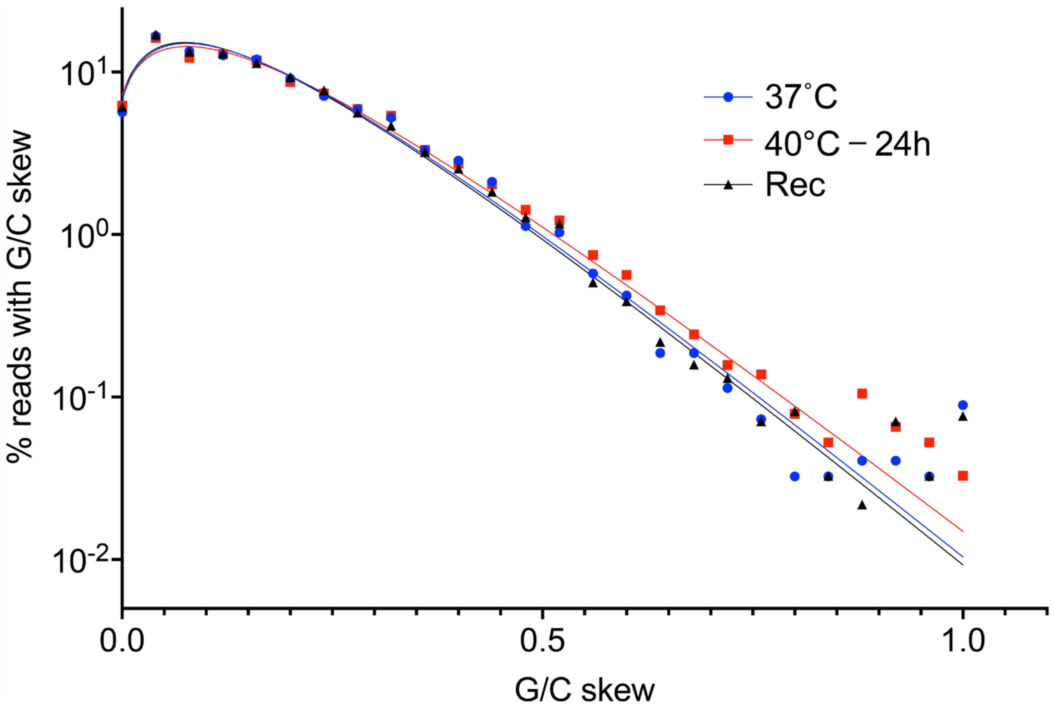

Supplementary Figure 2: Regions undergoing chimeric events upon heat stress show high G/C-skew in GM02639: The chimeric events at these higher G/C-skew regions are reparable and hence lost upon recovery post heat stress. The G/C-skew was calculated as (G − C)/(G + C). The data points were fitted to non-linear damped sine wave function with initial decay constant K = 4, lambda = 0.2 and phase shift = 0. The decay constant K values for GM02639 37°C, 40°C-24 h and Rec samples were 10.44, 9.959 and 10.57 respectively.

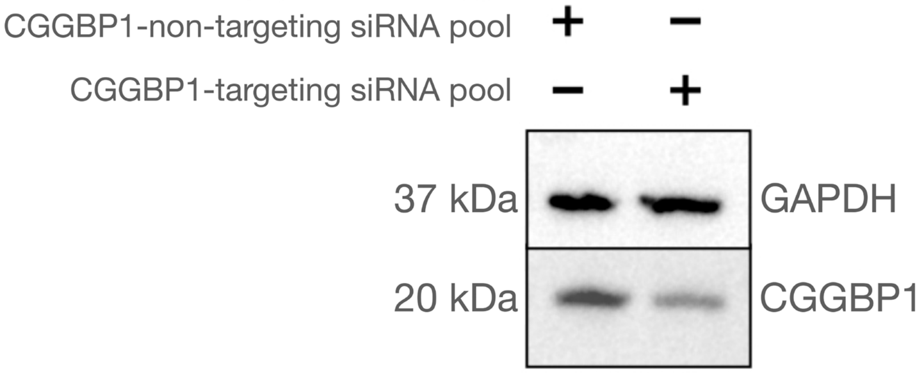

Supplementary Figure 3: Depletion of CGGBP1 by siRNA-mediated knockdown in GM01391: A near 50% knockdown of CGGBP1 was achieved by using CGGBP1-targeting siRNA pool. GAPDH is used as a loading control for the amount of protein. Such a partial knockdown of CGGBP1 in the fibroblasts allows studying the cells without a string cell cycle arrest and senescence-like phenotype.

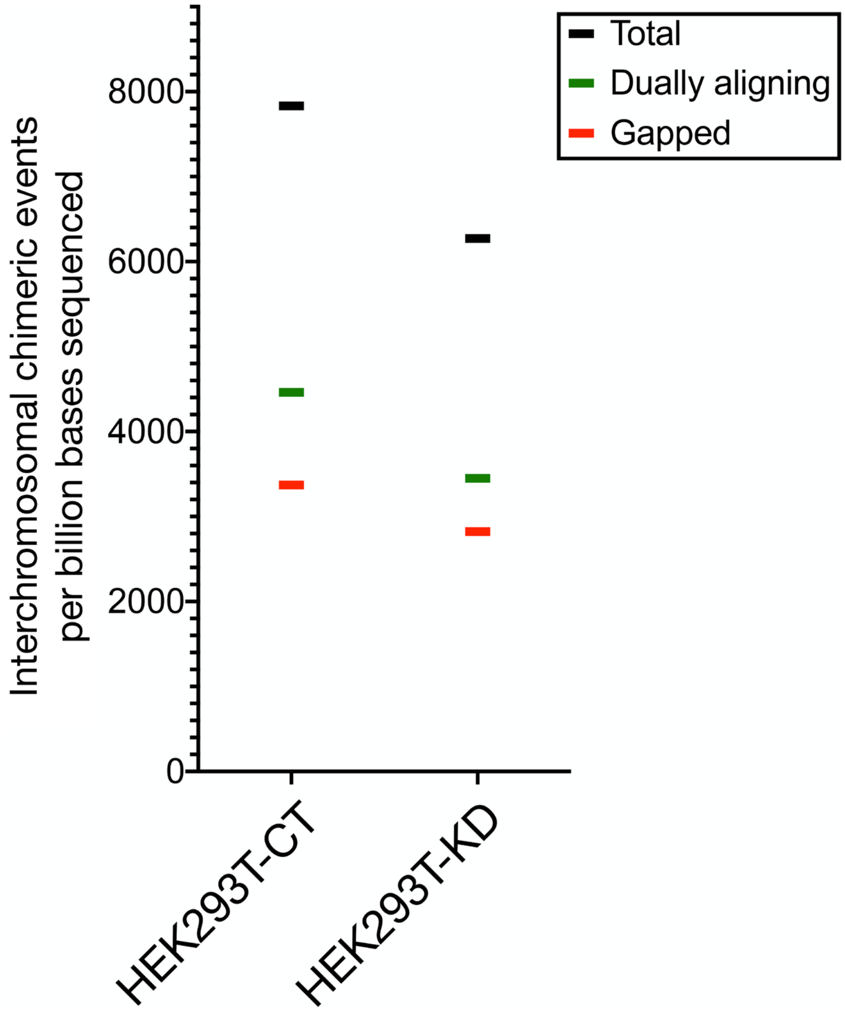

Supplementary Figure 4: Recovery from heat stress causes high occurrences of chimeric events in HEK293T cells. The total chimeric events in the Rec samples are enhanced to nearly two folds (compared to the respective samples in Figure 2A) with a proportionate increase in gapped and dually aligning chimeric events in CT as well as KD. The high levels of chimeric events in these cells were associated with high mortality upon recovery from heat stress.

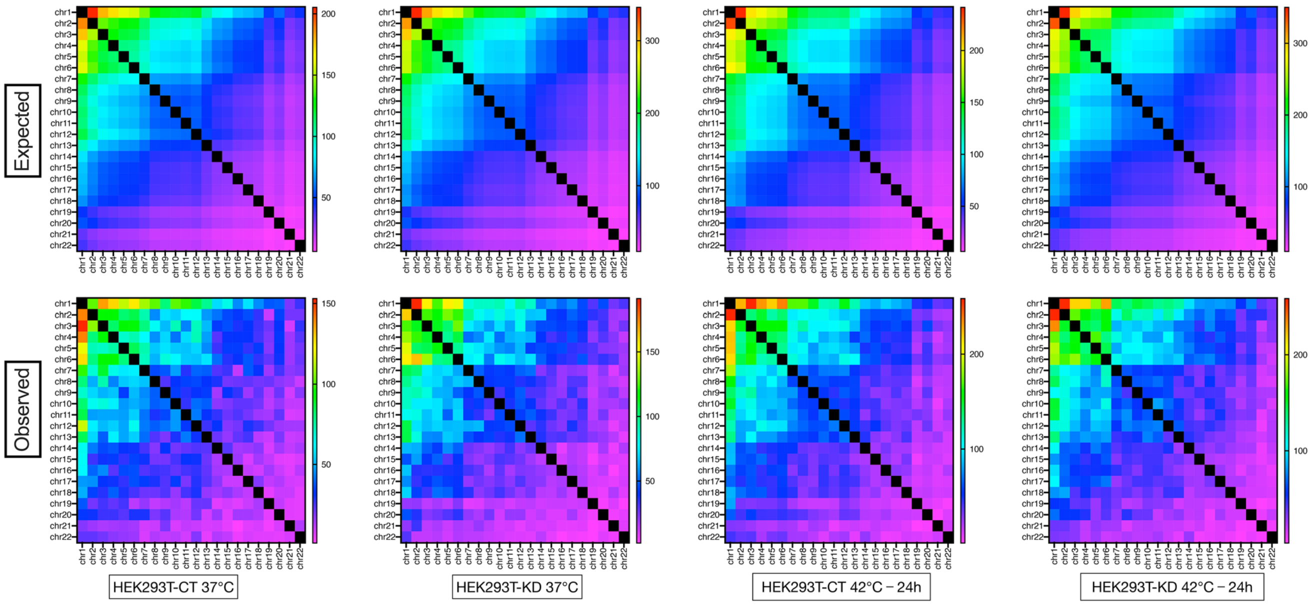

Supplementary Figure 5: Similar to GM02639 cells, in HEK293T cells also we observed a dependence of chimeric chromosomal DNA occurrence on chromosomal lengths thereby suggesting a random distribution of the chimeric events without any inter-chromosomal preferences: Like shown in Supplementary Figure 1, the expected chimeric events depict the distribution profiles if all the events were distributed evenly on the different autosomes proportional to their lengths. A strong dependence of chimeric event events on the chromosomal lengths is observed barring some inconsistent exceptions. In this analysis, the A-U-B events were considered non-directional and the A-U-B events were not differentiated from the B-U-A events.

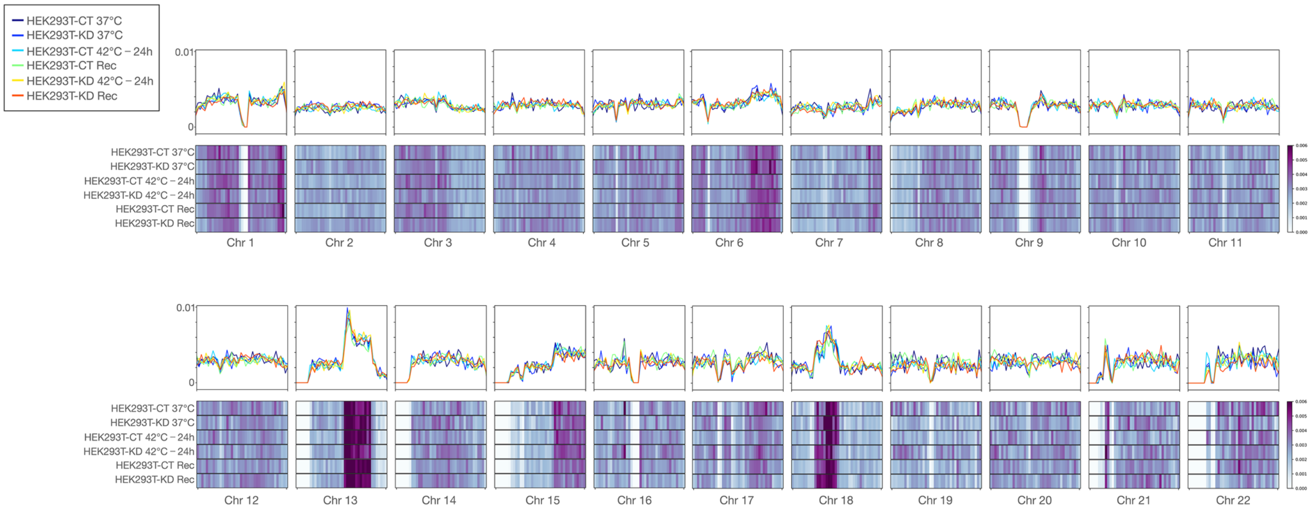

Supplementary Figure 6: No major intrachromosomal regional differences were observed between the various HEK293T samples in the distribution of chimeric events on the autosomes. The intra-chromosomal distributions of the chimeric events were calculated in a bin length of 5 kb. The profile plots in the top panels above the heatmaps have arbitrary units on the Y-axis. The X-axis has a scaled representation of all chromosomes to 0.2 Mb.

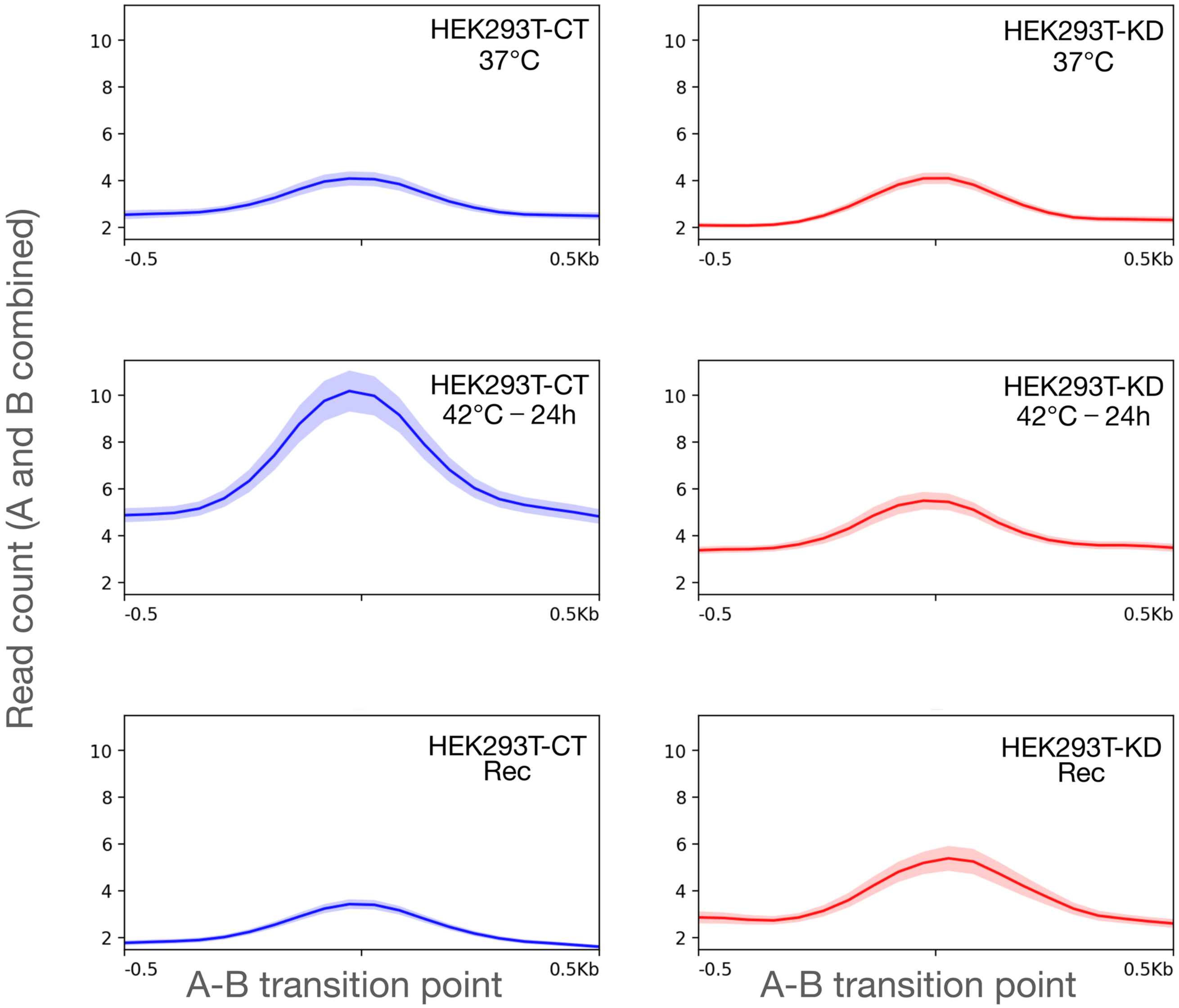

Supplementary Figure 7: The distribution of non-chimeric events at and in the immediate flanks of the chimeric events show that the chimeric events are not artefacts. The Y-axes represent the read count at the chimeric 0.2 kb bin centre. The Data are plotted for 0.5 kb upstream and downstream. The central enrichment of the non-chimeric reads indicates a non-uniform sequencing coverage in the region.

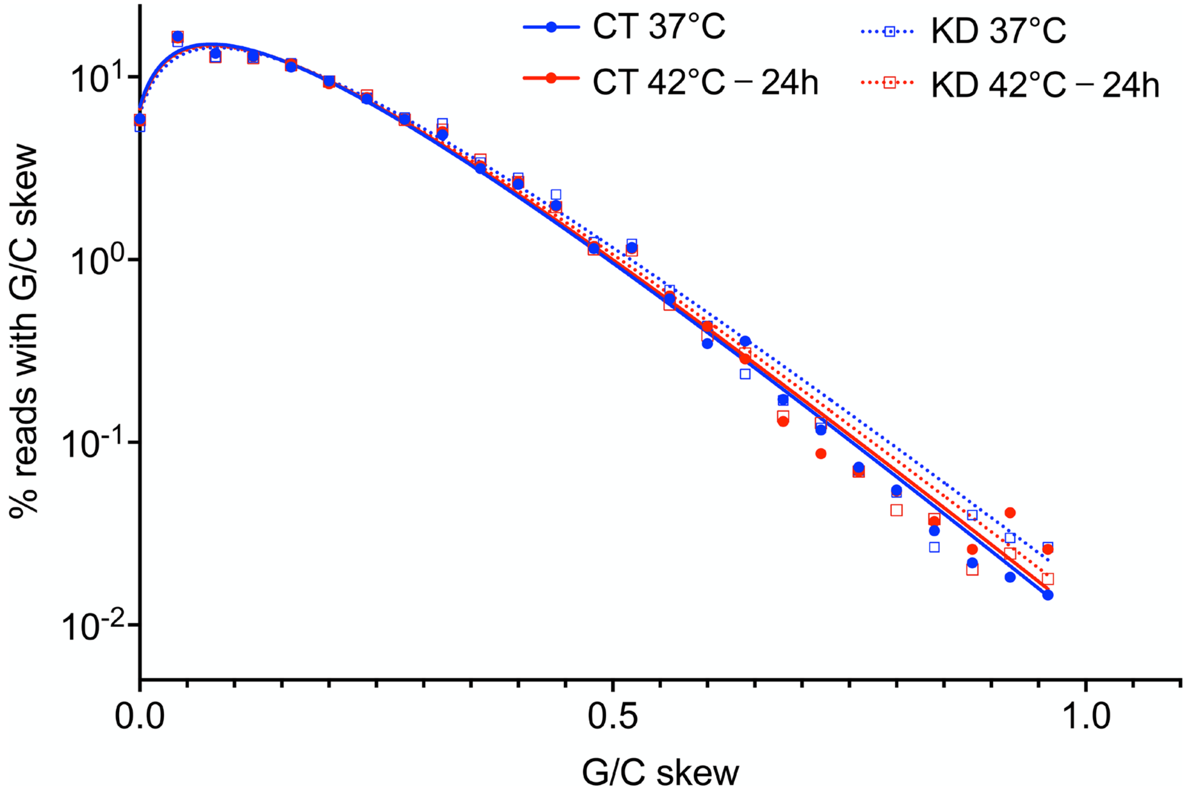

Supplementary Figure 8: Regions undergoing chimeric events in HEK293T upon heat stress show high G/C-skew: The chimeric events at these higher G/C-skew regions are reparable and hence lost upon recovery post heat stress. The G/C-skew was calculated as (G − C)/(G + C). The data points were fitted to non-linear damped sine wave function with initial decay constant K = 4, lambda = 0.2 and phase shift = 0. The decay constant K is highest for HEK293T-CT 37°C and lowest for HEK293T-KD 37°C.

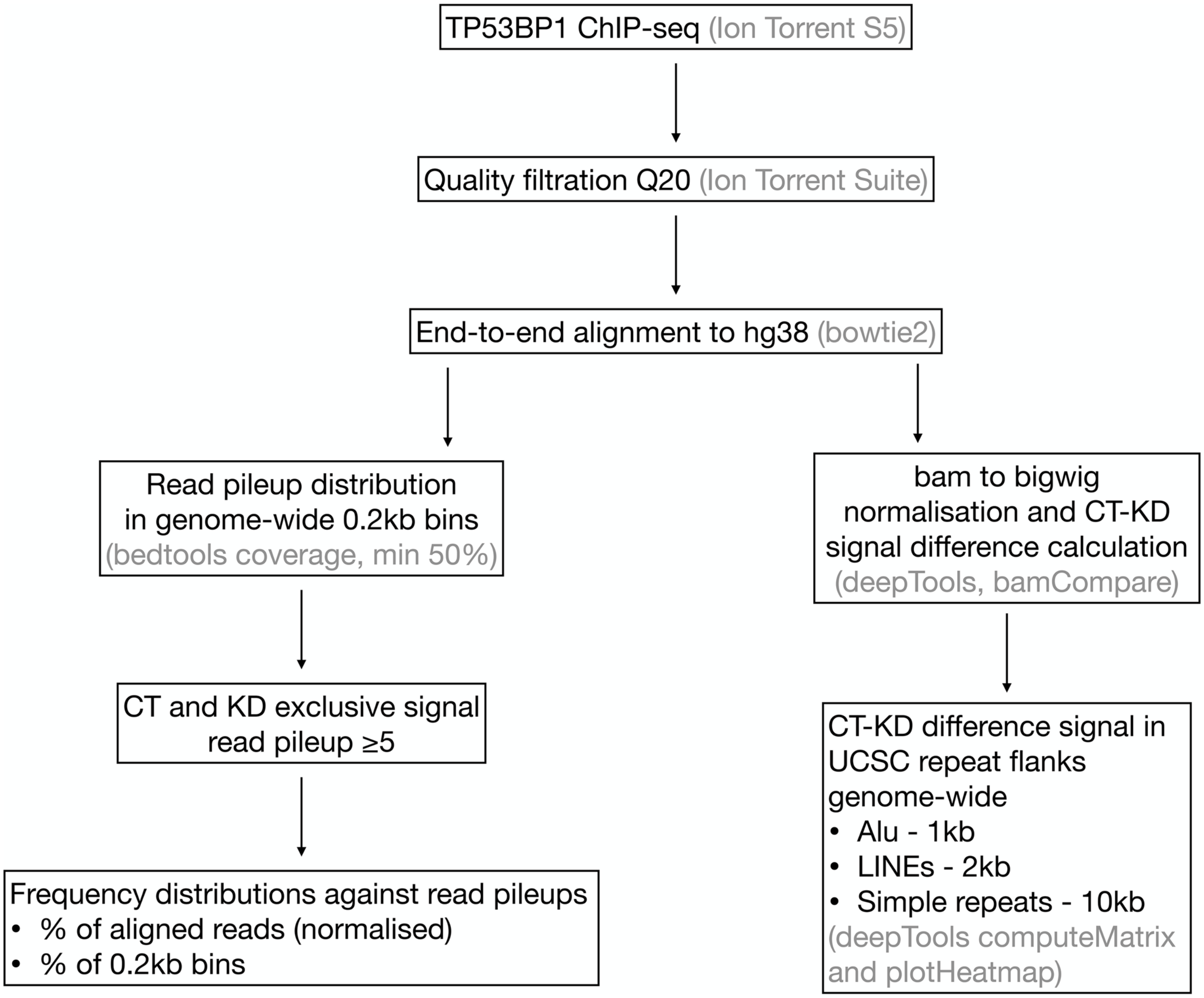

Supplementary Figure 9: Data analysis pipeline for the TP53BP1 ChIP-sequencing. The results of this pipeline are shown in Figure 3. The raw data files are available vide GSE169435.

Supplementary Table 1: GM02639 sequencing details at 37°C, 40°C-24 h and Rec

Fibroblast (GM02639) sequencing details (data run through Porechop)			
**Sample name**	**37°C**	**40°C-24 h**	**Rec**
Read count	597103	1528595	2349238
Base count	8003216090	3889197457	7184368217
Mean read length	13403.41	2544.30	3058.17
Reads mapped by bowtie2	572621	1285361	2010421
Aligned reads (%)	95.90	84.09	85.58

Supplementary Table 2: GM02639 repeat-content at 37°C, 40°C-24 h and Rec

Repeat types	37°C			40°C-24 h			Rec		
	Number of elements	Length occupied (bp)	Sequence (%)	Number of elements	Length occupied (bp)	Sequence (%)	Number of elements	Length occupied (bp)	Sequence (%)
**SINEs:**	1142	125687	7.28	2163	292474	9.6	2609	347859	10.96
**ALUs**	1067	119247	6.91	1992	276179	9.07	2462	334884	10.55
**MIRs**	74	6380	0.37	170	16141	0.53	146	12895	0.41
**LINEs:**	4093	700490	40.57	5046	800672	26.29	6567	1045142	32.93
**LINE1**	4025	694278	40.21	4928	788116	25.87	6404	1028639	32.41
**LINE2**	63	5770	0.33	105	11391	0.37	150	15266	0.48
**L3/CR1**	4	396	0.02	9	827	0.03	11	1067	0.03
**LTR elements:**	710	105263	6.1	1203	164224	5.39	1260	168221	5.3
**ERVL**	54	6570	0.38	149	17028	0.56	129	14192	0.45
**ERVL-MaLRs**	166	18397	1.07	397	47752	1.57	406	45705	1.44
**ERV_class I**	337	54013	3.13	510	75581	2.48	550	81453	2.57
**ERV_class II**	153	26283	1.52	139	23087	0.76	164	25964	0.82
**DNA elements:**	149	16608	0.96	330	35943	1.18	343	33572	1.06
**hAT-Charlie**	62	6633	0.38	126	12964	0.43	161	15661	0.49
**TcMar-Tigger**	58	6732	0.39	142	16926	0.56	133	13436	0.42
**Unclassified:**	384	51035	2.96	387	41448	1.36	501	55290	1.74
**Total interspersed repeats:**		999083	57.86		1334761	43.82		1650084	51.99
**Small RNA:**	174	11219	0.65	261	20524	0.67	266	14829	0.47
**Satellites:**	604	104211	6.04	1319	223112	7.32	1179	198216	6.25
**Simple repeats:**	571	23838	1.38	1087	64160	2.11	908	38319	1.21
**Low complexity:**	56	2872	0.17	128	7715	0.25	105	5887	0.19
**% of bases masked**		66.1			54.18			60.1	
**% of bases unmasked**		33.9			45.82			39.9	

Supplementary Table 3: GM02639 repeat-subfamilies at 37°C, 40°C-24 h and Rec

GM02639 samples	%Alu-SINEs			%LINE-1		
	**AluJ**	**AluS**	**AluY**	**L1H**	**L1M**	**L1P**
**37°C**	9.61	48.26	42.13	2.62	10.57	86.81
**40°C-24 h**	13.12	59.44	27.44	2.97	23.74	73.29
**Rec**	12.08	57.97	29.96	3.54	18.98	77.49

Supplementary Table 4: CpG and GC content in U bins of GM02639 at 37°C, 40°C-24 h and Rec

GM02639 samples	37°C	40°C-24 h	Rec
Number of U bins	8633	15230	15868
% CpG content	1.31	1.36	1.27
% GC content	41.00	41.71	40.98

Supplementary Table 5: GM02639-CT and GM02639-KD repeat-content

Repeat types	GM02639-CT			GM02639-KD		
	Number of elements	Length occupied (bp)	Sequence (%)	Number of elements	Length occupied (bp)	Sequence (%)
**SINEs:**	14917	1931519	20.41	6066	853679	19.58
**ALUs**	14375	1884855	19.92	5812	830905	19.06
**MIRs**	542	46664	0.49	254	22774	0.52
**LINEs:**	10123	1245583	13.16	3893	509551	11.69
**LINE1**	9441	1183032	12.5	3630	484201	11.1
**LINE2**	640	58961	0.62	251	24041	0.55
**L3/CR1**	33	2655	0.03	7	702	0.02
**LTR elements:**	4130	449761	4.75	1354	152978	3.51
**ERVL**	685	76136	0.8	212	22163	0.51
**ERVL-MaLRs**	1820	203026	2.15	609	71300	1.64
**ERV_class I**	1226	132615	1.4	459	51294	1.18
**ERV_class II**	382	36316	0.38	62	7004	0.16
**DNA elements:**	1316	125318	1.32	540	51774	1.19
**hAT-Charlie**	592	52865	0.56	235	20568	0.47
**TcMar-Tigger**	536	54842	0.58	216	23943	0.55
**Unclassified:**	1657	111204	1.18	674	47300	1.08
**Total interspersed repeats:**		3863385	40.83		1615282	37.05
**Small RNA:**	1572	88300	0.93	628	35470	0.81
**Satellites:**	11242	2087359	22.06	6102	1164495	26.71
**Simple repeats:**	6888	567282	6	4029	356982	8.19
**Low complexity:**	202	9405	0.1	73	3522	0.08

Supplementary Table 6: MeDIP reads with allelic identities in GM02639-CT and GM02639-KD

Samples	GM02639-CT	GM02639-KD
Read counts with unexpected allelic identities	2328783	1084232
Read counts with expected allelic identities	4237592	1998307
Somatic mutation rate	54.96	54.26

Supplementary Table 7: X chromosomal allelic identities in GM02639-CT and GM02639-KD

Samples	GM02639-CT	GM02639-KD
X chromosomal read count with maternal allelic identity	16548	7947
Total X chromosomal read count with non-maternal allelic identity	48160	21816
Total X chromosmal read count subjected to allele identification	64708	29763
Somatic mutation rate for Xc hromosome	25.57	26.70

Supplementary Table 8: Autosomal allelic identities and interallelic chimeras in GM02639-CT and GM02639-KD

Samples	GM02639-CT	GM02639-KD
Autosomal reads with maternal allelici dentity	816032	394787
Autosomal reads with paternal allelic identity	985983	466003
Autosomal reads with chimeric alleleic identity	76874	39370
Total autosomal reads subjected to allelic identification	1878889	900160
Inter allelic chimera frequency	4.09	4.37
Somatic mutation rate for autosomes	54.96	54.26
Corrected interallelic chimera frequency	1.84	2.00

Supplementary Table 9: Repeat content in GM02639-CT and GM02639-KD

Repeat types	GM02639-CT			GM02639-KD		
	Number of elements	Length occupied (bp)	Sequence (%)	Number of elements	Length occupied (bp)	Sequence (%)
**SINEs:**	4228	610798	16.54	2847	421569	16.77
**ALUs**	3761	557317	15.09	2517	385113	15.32
**MIRs**	465	53291	1.44	327	36130	1.44
**LINEs:**	3897	725424	19.65	2543	465694	18.52
**LINE1**	3385	656389	17.78	2178	420403	16.72
**LINE2**	467	62928	1.7	332	41635	1.66
**L3/CR1**	29	4326	0.12	26	3085	0.12
**LTR elements:**	1721	298335	8.08	1108	193987	7.72
**ERVL**	312	53566	1.45	193	34211	1.36
**ERVL-MaLRs**	686	112482	3.05	467	75439	3
**ERV_class I**	612	111808	3.03	385	72928	2.9
**ERV_class II**	79	16387	0.44	39	8458	0.34
**DNA elements:**	696	96124	2.6	501	68906	2.74
**hAT-Charlie**	329	41496	1.12	230	30269	1.2
**TcMar-Tigger**	216	34349	0.93	166	25275	1.01
**Unclassified:**	28	2292	0.06	19	1721	0.07
**Total interspersed repeats:**		1732973	46.93		1151877	45.82
**Small RNA:**	25	1893	0.05	15	1247	0.05
**Satellites:**	842	187698	5.08	474	103459	4.12
**Simple repeats:**	878	35996	0.97	585	23688	0.94
**Low complexity:**	107	4911	0.13	69	3201	0.13

Supplementary Table 10: X-U-A and Y-U-A chimeric events in GM02639-CT and GM02639-KD

Samples	GM02639-CT	GM02639-KD
Total chimeric reads	44026	19734
Reads with X-U-A chimeras	2206	941
Reads with Y-U-A chimeras	3046	1848
X-U-A (%)	5.01	4.77
Y-U-A (%)	6.92	9.36

Supplementary Table 11: Sequencing details of GM01391-CT and GM01391-KD

Sample name	GM01391-CT	GM01391-KD
Read count	104768797	117629570
Base count	15447113744	17535893169
Mean read length	147.44	149.08
Reads mapped by bowtie2	67725152	80980111
% mapped reads	64.64	68.84

Supplementary Table 12: MeDIP reads with allelic identities in GM01391-CT and GM01391-KD

Samples	GM01391-CT	GM01391-KD
Read counts with unexpected allelic identities	6799763	2354827
Read counts with expected allelic identities	11509946	4471689
Somatic mutation rate	59.08	52.66

Supplementary Table 13: MeDIP reads with allelic identities and interallelic chimeras in GM02639-CT, GM02639-KD, GM01391-CT and GM01391-KD

Samples	GM02639-CT	GM02639-KD	GM01391-CT	GM01391-KD
Reads with maternal allelic identity	816032	394787	2053554	940277
Reads with paternal allelic identity	985983	466003	2376579	1002715
Interallelic chimeric read count	76874	39370	75879	108513
Reads subjected to allelic identification	1878889	900160	4506012	2051505
Non-chimeric reads per interallelic chimeric event	23.4	21.9	58.4	17.9

Supplementary Table 14: Normalized number of MeDIP reads with interallelic chimeras in GM02639-CT, GM02639-KD, GM01391-CT and GM01391-KD

Samples	Reads with inter allelic chimera (without normalization)	Reads with inter allelic chimera (after normalization)
GM02639-CT	76874	37561
GM02639-KD	39370	39370
GM01391-CT	75879	75879
GM01391-KD	108513	90751

The normalization was done by randomly selecting the same number of reads (before variant calling) from GM02639-CT and GM01391-KD to match the read counts of GM02639-KD, GM01391-CT respectively. The interallelic chimeras were identified again with the normalized read sets.

Supplementary Table 15: ANOVA test details of repeat types detected in U bins HEK293T-CT and HEK293T-KD at 37°C, 42°C-24 h and Rec. See Supplementary Table 15

Supplementary Table 16: TP53BP1 ChIP sequencing details and A-U-B chimeric events in ChIP-sequencing datasets for HEK293T-CT and HEK293T-KD

Sample name	HEK293T-CT	HEK293T-KD
Read count	90638165	83117053
Base count	13866969515	12314145819
Mean read length	152.99	148.15
Reads mapped by bowtie2	49198728	31046804
Reads remained unmapped	41439437	52070249
Base count for unmapped reads	5938568698	7395489494
Total A-U-B chimeric events in unmapped reads	4888	5472
Chimeric DNA events per billion bases sequenced	823.09	739.91

Supplementary Table 17: Repeat content in U bins of TP53BP1 ChIP-seq data from HEK293T-CT and HEK293T-KD

Repeat types	HEK293T-CT			HEK293T-KD		
	Number of elements	Length occupied (bp)	Sequence (%)	Number of elements	Length occupied (bp)	Sequence (%)
**SINEs:**	2563	381682	43.21	1992	297479	29.63
**ALUs**	2546	380050	43.02	1938	291328	29.02
**MIRs**	17	1632	0.18	29	4263	0.42
**LINEs:**	740	92899	10.52	1046	126105	12.56
**LINE1**	732	92378	10.46	987	120840	12.04
**LINE2**	8	521	0.06	57	5087	0.51
**L3/CR1**	0	0	0	0	0	0
**LTR elements:**	215	35041	3.97	525	57657	5.74
**ERVL**	3	486	0.06	76	7344	0.73
**ERVL-MaLRs**	161	26678	3.02	221	24152	2.41
**ERV_class I**	42	6454	0.73	219	24633	2.45
**ERV_class II**	9	1423	0.16	7	1194	0.12
**DNA elements:**	51	3845	0.44	83	6308	0.63
**hAT-Charlie**	5	540	0.06	29	2203	0.22
**TcMar-Tigger**	23	1761	0.2	38	3100	0.31
**Unclassified:**	40	3089	0.35	82	4828	0.48
**Total interspersed repeats:**		516556	58.48		492377	49.05
**Small RNA:**	149	8425	0.95	112	7407	0.74
**Satellites:**	987	171056	19.36	1035	160049	15.94
**Simple repeats:**	757	62855	7.12	520	44163	4.4
**Low complexity:**	12	718	0.08	7	353	0.04
