# Supplementary material for: Chimeric chromosome landscapes of human somatic cell cultures show dependence on stress and regulation of genomic repeats by CGGBP1
Source: Oncotarget. 2022 Jan 17;13:136–55. doi: 10.18632/oncotarget.28174 (PMC8765472; doi:10.18632/oncotarget.28174)
Supplement: Supplementary file 2 [file oncotarget-13-28174-s002.pdf]

Supplementary Table 15: ANOVA test details of repeat types detected in U bins HEK293T-CT and HEK293T-KD at 37°C, 42°C-24 h and Rec.

Repeated measures ANOVA summary	Repeat-free (Fig 2D)				
Assume sphericity?	Yes				
F	88.9				
P value	<0.0001				
P value summary	****				
Statistically significant (P < 0.05)?	Yes				
R squared	0.6897				
Was the matching effective?					
F	103.6				
P value	<0.0001				
P value summary	****				
Is there significant matching (P < 0.05)?	Yes				
R squared	0.8654				
ANOVA table	SS	DF	MS	F (DFn, DFd)	P value
Treatment (between columns)	4.379	5	0.8759	F (5, 200) = 88.90	P<0.0001
Individual (between rows)	40.81	40	1.02	F (40, 200) = 103.6	P<0.0001
Residual (random)	1.97	200	0.009852		
Total	47.16	245			
Data summary					
Number of treatments (columns)	6				
Number of subjects (rows)	41				
Number of missing values	0				

Repeated measures ANOVA summary	Alu-SINEs (Fig 2E)				
Assume sphericity?	Yes				
F	21.85				
P value	<0.0001				
P value summary	****				
Statistically significant (P < 0.05)?	Yes				
R squared	0.3533				
Was the matching effective?					
F	32.55				
P value	<0.0001				
P value summary	****				
Is there significant matching (P < 0.05)?	Yes				
R squared	0.808				
ANOVA table	SS	DF	MS	F (DFn, DFd)	P value
Treatment (between columns)	3.23	5	0.6461	F (5, 200) = 21.85	P<0.0001
Individual (between rows)	38.49	40	0.9623	F (40, 200) = 32.55	P<0.0001
Residual (random)	5.913	200	0.02957		
Total	47.64	245			
Data summary					
Number of treatments (columns)	6				
Number of subjects (rows)	41				
Number of missing values	0				

Repeated measures ANOVA summary	LINEs (Fig 2F)				
Assume sphericity?	Yes				
F	22.95				
P value	<0.0001				
P value summary	****				
Statistically significant (P < 0.05)?	Yes				
R squared	0.3646				
Was the matching effective?					
F	40.59				
P value	<0.0001				
P value summary	****				
Is there significant matching (P < 0.05)?	Yes				
R squared	0.8376				
ANOVA table	SS	DF	MS	F (DFn, DFd)	P value
Treatment (between columns)	2.253	5	0.4505	F (5, 200) = 22.95	P<0.0001
Individual (between rows)	31.86	40	0.7965	F (40, 200) = 40.59	P<0.0001
Residual (random)	3.925	200	0.01963		
Total	38.04	245			
Data summary					
Number of treatments (columns)	6				
Number of subjects (rows)	41				
Number of missing values	0				

Repeated measures ANOVA summary	Simple repeats (Fig 2G)				
Assume sphericity?	Yes				
F	17.59				
P value	<0.0001				
P value summary	****				
Statistically significant (P < 0.05)?	Yes				
R squared	0.3054				
Was the matching effective?					
F	17.95				
P value	<0.0001				
P value summary	****				
Is there significant matching (P < 0.05)?	Yes				
R squared	0.7137				
ANOVA table	SS	DF	MS	F (DFn, DFd)	P value
Treatment (between columns)	4.614	5	0.9228	F (5, 200) = 17.59	P<0.0001
Individual (between rows)	37.67	40	0.9417	F (40, 200) = 17.95	P<0.0001
Residual (random)	10.49	200	0.05247		
Total	52.78	245			
Data summary					
Number of treatments (columns)	6				
Number of subjects (rows)	41				
Number of missing values	0				
